# Detecting and Evaluating Urban Clusters with Spatiotemporal Big Data

**DOI:** 10.3390/s19030461

**Published:** 2019-01-23

**Authors:** Luliang Tang, Jie Gao, Chang Ren, Xia Zhang, Xue Yang, Zihan Kan

**Affiliations:** 1 State Key Laboratory of Information Engineering in Surveying, Mapping, and Remote Sensing, Wuhan University, Wuhan 430079, China; tll@whu.edu.cn (L.T.); kzh@whu.edu.cn (Z.K.); 2 School of Urban Design, Wuhan University, Wuhan 430070, China; xiazhang@whu.edu.cn; 3 Faculty of Information Engineering, China University of Geosciences, Wuhan 430074, China; yangxue@cug.edu.cn

**Keywords:** urban clusters, clustering, rationality, conformance, travel activities, spatiotemporal big data

## Abstract

The design of urban clusters has played an important role in urban planning, but realizing the construction of these urban plans is quite a long process. Hence, how the progress is evaluated is significant for urban managers in the process of urban construction. Traditional methods for detecting urban clusters are inaccurate since the raw data is generally collected from small sample questionnaires of resident trips rather than large-scale studies. Spatiotemporal big data provides a new lens for understanding urban clusters in a natural and fine-grained way. In this article, we propose a novel method for Detecting and Evaluating Urban Clusters (DEUC) with taxi trajectories and Sina Weibo check-in data. Firstly, DEUC applies an agglomerative hierarchical clustering method to detect urban clusters based on the similarities in the daily travel space of urban residents. Secondly, DEUC infers resident demands for land-use functions using a naïve Bayes’ theorem, and three indicators are adopted to assess the rationality of land-use functions in the detected clusters—namely, cross-regional travel index, commuting direction index, and fulfilled demand index. Thirdly, DEUC evaluates the progress of urban cluster construction by calculating a proposed conformance indicator. In the case study, we applied our method to detect and analyze urban clusters in Wuhan, China in the years 2009, 2014, and 2015. The results suggest the effectiveness of the proposed method, which can provide a scientific basis for urban construction.

## 1. Introduction

Urban clusters significantly reduce traffic through a mixed agglomeration of various land-use functions [1,2,3,4]. Specifically, a land-use multifunctional cluster in a clustered city has relatively well-developed infrastructure and residential facilities [5], meeting the needs of the majority of residents in the cluster, thus reducing cross-regional travels [1,2,3,4]. Xu et al. [6] found that a small activity space was enough to fulfill the demands of the majority of residents in Shenzhen, China, consistent with the municipal government’s goal to achieve a clustered city. Based on these advantages, the design of urban clusters has become an active area of urban planning.

Realizing the construction of urban clusters is a long process, so it is indispensable for urban managers to assess the construction’s progress. Traditional methods are inaccurate since the detection of urban clusters generally relies on small sample questionnaires of resident trips [1,7], which are easily influenced by questionnaire design and subjective judgements. Spatiotemporal big data, such as vehicle trajectories [8,9,10], cellphone data [11,12], social media data [13,14,15], and geo-tagged photos [16,17], provides first-hand information about individual activities, which can complement traditional methods [18] and provide a new lens for understanding cities [19]. Human behavior, as documented by digital trails, follows inherent regularity [20], which can be applied to the study of land use and urban space [9,12,21]. Furthermore, the application of Geographic Information Systems (GIS) tools provides opportunities to study multiple geographical scales from the individual to the urban area, which can facilitate the study of reciprocal interactions between people and cities [22,23]. In this article, we combine spatiotemporal attributes of taxi GPS data and geo-semantic information of Sina Weibo check-in data to detect and evaluate urban clusters.

The remainder of this article is structured as follows. Section 2 introduces some related work about land-use studies. In Section 3, we describe the Detecting and Evaluating Urban Clusters (DEUC) method and its algorithm in detail. In Section 4, we apply our method for detecting and analyzing urban clusters in Wuhan, China, as a case study. The conclusions are shown in Section 5.

## 2. Related Work

Spatiotemporal big data, containing information about resident activities and reciprocal interactions between residents and cities, has been widely applied in urban land-use studies [9,13,24]. Moreover, the development of spatial information technologies, such as geographic information system and remote sensing, provides technical support for relevant studies [25,26,27]. The retrieval of current land-use studies can be divided into two stages: detection of resident activities and delineation of land-use patterns based on resident activities [28].

In the first stage, the spatiotemporal big data has opened up new horizons for studying various aspects of resident activities. For instance, Widhalm et al. [11] proposed a probability method in order to extract daily activities from cellphone data. Gong et al. [29] inferred trip purposes and discovered travel patterns from taxi trajectories. In addition, the popularity of the Internet allows users to upload their geographic locations via mobile communication devices. The location data generated in this way is called check-in data [13]. The check-in data is rich in geo-semantic information and has been widely applied in exploring the spatiotemporal patterns of resident activities [13,14,15]. For instance, Tu et al. [14] explored diurnal patterns of urban functions by analyzing resident activities inferred from cellphone data and check-in data. Resident activities containing socioeconomic information closely related to land use can be applied to detect land-use patterns.

Therefore, in the second stage, using the abundant socioeconomic information derived from resident activities, researchers classified land-use types and investigated new land-use patterns related to resident activities. For instance, Soto et al. [12,30] applied a fuzzy-c means method to extract land-use types automatically (including office, business, nightlife, leisure, and residential areas). Pei et al. [28] used a semi-supervised classification method to classify urban land into residential, business, commercial, open space, and others. Frias-Martinez et al. [31] detected land uses and identified urban Points of Interest (POI) automatically from Twitter data. Toole et al. [32] applied a random forest method to infer urban land use, with weekday–weekend cellphone data. Moreover, some scholars began to integrate multi-sourced data for land-use research [33,34,35,36]. Similarly to that combined building-level social media data with remote sensing images, Chen et al. [33] used a k-medoids clustering method to delineate urban functional areas. The relevant research even extended to the field of indoor environments. For example, based on MIT’s Wi-Fi data, Calabrese et al. [25] used a k-means clustering method to match physical environments to corresponding activities, such as the lab and classroom. Some recent land-use studies with spatiotemporal big data are listed in Table 1, indicating that although researchers have done a great deal of work on land use, there are two problems worth further study.

The first problem is that these studies mainly apply spatiotemporal big data to detect land-use structures from the perspective of functional zones, but they lack the detection of the comprehensive, clustered urban layout. The second is that they lack the support of evaluation of the urban construction progress. Therefore, we propose a DEUC method with spatiotemporal big data, in which we detect urban clusters from resident travel activities, analyze the rationality of land-use functions in the detected clusters, and evaluate conformance between detected urban clusters and that in urban planning documents.

## 3. Methodology

In this section, we present our DEUC method. Using taxi GPS data and Weibo check-in data, this method consists of two main components: the detection (Section 3.1) and the quantitative evaluation (Section 3.2) of urban clusters. We superimposed the study areas with suitable grid cells (Section 3.1.1). The locations of Pick-Up Points (PUPs) and Drop-Off Points (DOPs) extracted from taxi GPS data were regarded as the origins and destinations of resident trips. By measuring the spatial distribution of Origin-Destinations (ODs) originating from each grid cell, the daily travel space of urban residents in each grid cell can be acquired, which is the basis for clustering (Section 3.1.2). We grouped the grid cells with similar travel space into clusters in order to detect urban clusters (Section 3.1.3). Through combination of the spatiotemporal attributes of taxi GPS data with geo-semantic information derived from Weibo check-in data, resident demands for land-use functions can be acquired by inferring their daily activity types and used for analyzing the rationality of land-use functions in the detected clusters (Section 3.2.1). The travel behaviors of residents straightforwardly reflect whether the land-use functions are reasonable. Hence, we adopted three indicators—namely, the cross-regional travel index, commuting direction index, and fulfilled demand index, to analyze the travel behaviors of residents and quantitatively evaluate land-use functions (Section 3.2.2). Moreover, we developed an indicator to evaluate conformance between the detected results and planned clusters, which is helpful when evaluating the progress of urban cluster construction (Section 3.2.3). The following two sections describe each step in detail.

### 3.1. Detection of Urban Clusters from Daily Travel Space of Urban Residents

#### 3.1.1. Determining the Grid Cell Size

At the beginning of the DEUC method, the study area was superimposed with regular grid cells, as widely applied in movement analysis and modelling [6,37,38]. For simplification, we assumed that resident activities would occur in the same grid cell where PUPs and DOPs are located. As PUP and DOP of a taxi trip are generally as close as possible to the place where the activity occurs, the location of PUP/DOP should be within walking distance from where the activity takes place. Hence, we regarded grid cells as “walkable grid cells”, and the longest distance (the diagonal distance) within a cell should not exceed the threshold of suitable walking distance. In the relevant research, the distance of a 10 min walk (around 750 m) is regarded as the threshold for suitable walking distance [39,40]. Hence, we chose 500 m as the grid cell size.

#### 3.1.2. Measuring Daily Travel Space of Urban Residents

In general, the number of resident trips will decrease as travel distance increases. Therefore, we assumed that the DOPs of trips originating from the same grid cell would roughly match a normal spatial distribution. Standard Deviational Ellipses (SDE) are widely used to measure the spatial distribution of a group of points with normal spatial distribution [41,42]; hence, we applied SDEs to measure the daily travel space of urban residents. The farther a trip is, the more random the travel, which means a smaller weight should be assigned to the SDE when the trip distance is far. Therefore, before constructing SDEs, we set different weights ($W^{\prime }$) for each DOP based on the trip distance, as shown in Equation (1):

(1) $$W=1/\sqrt{{\left(X_{DOP}-X_{PUP}\right)}^{2}+{\left(Y_{DOP}-Y_{PUP}\right)}^{2}}$$

where $\left(X_{PUP},Y_{PUP}\right)$ and $\left(X_{DOP},Y_{DOP}\right)$ denote coordinates of PUP/DOP, and *W* is the computed weight. All of the obtained weights are then normalized as $W^{\prime }$.

#### 3.1.3. Detecting Urban Clusters

A land-use multifunctional cluster can meet the daily travel requirements of the majority of residents in the cluster, thus reducing cross-regional travels. Therefore, a land-use multifunctional cluster can be considered as the daily travel space of residents within that cluster. Based on this assumption, urban clusters can be detected by grouping grid cells with similar SDEs, as described later in this section. However, the number and centers of clusters are unknown before grouping. In this case, using unsupervised classification is helpful to discover new knowledge [43]. Hence, we used the Agglomerative Hierarchical Clustering (AHC) method [37,43,44] in this study.

The grid cells are clustered based on the similarity of travel space of trips originating from each grid cell, which can be measured with the area of overlap of SDEs; the larger the overlapping area, the higher the similarity coefficient. Since grid cells are the basic unit for analysis, all the SDEs were projected on the divided grid cells. The corresponding SDE of each grid cell is represented by a grid cell set $A(g_{1},g_{2},\ldots ,g_{m})$, where $g_{m}$ denotes the grid cell number. In this step, the similarity measurement between any two SDEs then converts into the similarity measurement between two sets of grid cells. The similarity thus can be calculated by using Intersection over Union (also called the Jaccard coefficient), as shown in Equation (2). The Jaccard coefficient is obtained by dividing the intersection of two grid cell sets by the size of the union of the two grid cell sets [45]. Equation (2) indicates that the Jaccard coefficient is positively related with the ratio of overlap of the two grid cell sets:

(2) $$J(A,B)=\frac{\left|A\cap B\right|}{\left|A\cup B\right|}$$

where *A* and *B* denote the grid cell set $A(g_{1},g_{2},\ldots ,g_{m})$ and $B(g_{1}^{\prime },g_{2}^{\prime },\ldots ,g_{m}^{\prime })$, respectively. 

In the clustering process, we applied average linkage strategy. Specifically, the similarity between any two clusters can be computed as the average value of Jaccard coefficients between grid cell sets from the first cluster and grid cell sets from the second cluster. The two clusters with the highest average value are merged into a new cluster systematically. The clustering process runs until it reaches the optimal clustering number. In an optimal clustering result, the correlation inside each cluster must be stronger than the correlation between different clusters, as indicated by taxi trips. In detail, when the number of ODs with origins and destinations both in each cluster is larger than the number between it and any other clusters, the corresponding clustering count is considered as the optimal clustering number. In addition, a small number of ODs originating from a grid cell are likely to form several small clusters containing only themselves or containing very few grid cells. These outliers must be removed during the clustering process.

### 3.2. Quantitative Analysis and Evaluation of Urban Clusters

#### 3.2.1. Acquiring Resident Demands for Land-Use Functions Based on Inferred Activity Types

The preceding activity types of PUPs and succeeding activity types of DOPs can reflect the resident demands for land use types. Hence, we can acquire the demands for land uses by inferring activity types. In this method, we focus on five types of daily activity. The mapping relationships between the types of POIs and the activity types are listed in Table 2.

Based on Table 2, each piece of check-in data can be labeled as one type of activity according to their POIs. Let $C=\{y_{1},y_{2},\ldots ,y_{5}\}$, where *C* is the activity type set and $y_{1}-y_{5}$ denotes each activity type in Table 2. We inferred the preceding activity types of PUPs and succeeding activity types of DOPs based on the Bayes’ theorem, shown in Equation (3):

(3) $$P(y_{i}|x)=\frac{P(x|y_{i})P(y_{i})}{P(x)}$$

where *P(y_i_|x)* is a conditional probability representing the occurring probability of *y_i_*-type activity, given that *x* has occurred. Let $x=\{a_{1},a_{2},a_{3}\}$ be an activity to be inferred, and each element *a_i_* in *x* represents a feature:

- $a_{1}=$ {1, 2, 3,…, *n*} is the well-divided gird set in which *n* denotes the grid cell number.
- $a_{2}=$ {0:00–8:00, 8:00–10:00, 10:00–12:00, 12:00–14:00, 14:00–16:00, 16:00–18:00, 18:00–20:00, 20:00–22:00, 22:00–24:00} is the timeline in which the day is divided into nine intervals.
- $a_{3}=$ {workdays, weekends} is the day set.

By aggregating check-in data within each grid cell, the occurring probability of each feature *a_i_* given that *y_i_*-type activity has occurred, i.e., $P(a_{1}|y_{i})$, $P(a_{2}|y_{i})$, and $P(a_{3}|y_{i})$, can be computed. With the prior knowledge of activities from the check-in data, we could infer activity types related to PUPs and DOPs. We assumed that each feature was conditionally independent, and thus the computation of $P(y_{i}|x)$ could be simplified to Equation (4). If $P(y_{k}|x)=\max\{P(y_{1}|x),P(y_{2}|x),\ldots ,P(y_{5}|x)\}$, then $x\in y_{k}$. After the inference, we could match the corresponding demand for land-use functions (including residential, education, commercial, recreation, and business) based on the inferred activity types.

(4) $$P(x|y_{i})P(y_{i})=P(a_{1}|y_{i})P(a_{2}|y_{i})P(a_{3}|y_{i})P(y_{i})=P(y_{i})\prod_{j=1}^{3}P(a_{j}|y_{i})$$

#### 3.2.2. Evaluating the Rationality of Land-Use Functions in the Detected Clusters

In this section, we present how we adopted three indicators to analyze trips quantitatively, evaluating the rationality of land-use functions in the detected clusters.

**Definition** **1.** *(Cross-regional travel index p_ij_): The cross-regional travel index p_ij_ represents the proportion of ODs from cluster i to cluster j in the total ODs originating from cluster i. The ranges of i and j are determined by the clustering results:*

(5) $$p_{ij}=\frac{T_{ij}}{\sum_{m=1}^{k}T_{im}^{}}$$

*where T_ij_ denotes the number of ODs from cluster i to cluster j; k denotes the number of detected clusters; and $\sum_{m=1}^{k}T_{im}^{}$ denotes the number of ODs originating from cluster i. When i = j, p_ij_ is an internal travel index. The higher the value of the internal travel index, the more the internal land-use functions can fulfill the travel demands, suggesting that land-use functions in cluster i are more reasonable. When i ≠ j, this indicator reflects the external travel destinations of cluster i; the cluster j, corresponding to the highest value, is the main external travel destination.*

**Definition** **2.** *(Commuting direction index k_ij_): Commuting is an important part of resident daily trips. Commuting behaviors can reflect the jobs–housing balance. The commuting direction index k_ij_ is shown in Equation (6) [1], where k_ij_ is defined as the ratio of the difference between the number of commuting ODs from cluster i to cluster j to the sum of the number of commuting ODs from cluster i to cluster j and that from cluster j to cluster i:*

(6) $$k_{ij}=\frac{T_{ij}}{T_{ij}+T_{ji}}$$

*where k_ij_ >0.5 represents that the commuting pattern is from cluster i to cluster j, while k_ij_ <0.5 represents that the commuting pattern is from cluster j to cluster i. The more the value of k_ij_ deviates from 0.5, the more unbalanced the commuting pattern.*

**Definition** **3.** *(Fulfilled demand index d_iy_): The fulfilled demand index d_iy_ represents the proportion of ODs with both origins and destinations in cluster i among the total ODs originating from cluster i based on the y-type land-use demands:*

(7) $$d_{iy}=\frac{T_{ii}{}^{y}}{\sum_{m=1}^{k}T_{im}^{y}}$$

*where $\sum_{m=1}^{k}T_{im}^{y}$ denotes the number of ODs originating from cluster i based on the y-type land-use demands. The higher the value of d_iy_ is, the more the internal y-type land-use functions fulfill the demands, suggesting that y-type land-use function in cluster i is more reasonable.*

#### 3.2.3. Evaluating Conformance between Detected Clusters and Planned Clusters

In this section, we develop a Conformance Ratio (*CR*) to evaluate conformance between detected results and planned clusters in order to assess the progress of urban construction. The computation of *CR* is shown in Equation (8), in which the proportion of the area of the planned cluster *i* in the total area of all planned clusters is assigned as the weight. The higher the *CR*, the closer the detected result is to the planned urban clusters:

(8) $$CR=\sum_{i=1}^{k}(\frac{G_{detected}^{i}\cap G_{planned}^{i}}{G_{planned}^{i}}\times \frac{G_{planned}^{i}}{G_{planned}^{}})$$

where *G^i^_detected_* denotes the grid cells overlapped by detected cluster *i*; *G^i^_planned_* denotes the grid cells overlapped by the corresponding planned cluster *i*; *G_planned_* denotes the grid cells overlapped by all planned clusters; and *k* denotes the number of detected clusters.

## 4. Implementation and Results

### 4.1. Study Area and Dataset

**Figure 1:**
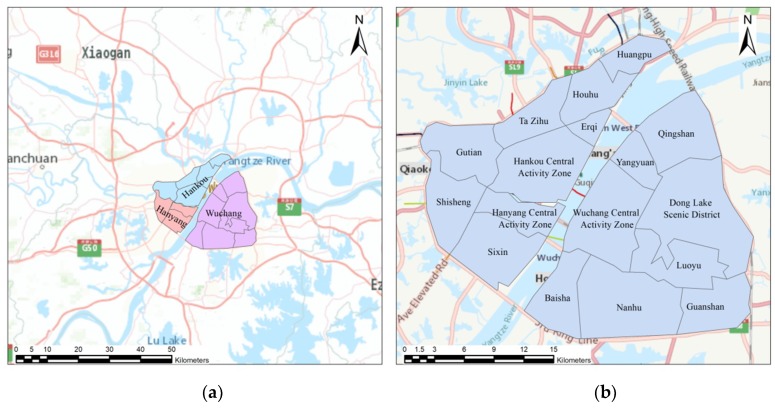
Study area: (**a**) the geographical location of the three towns of Wuhan in the map of China; (**b**) the planned urban clusters of Wuhan.

In this article, we selected the area inside the third ring expressway in Wuhan, China, as the study area. Wuhan is one of the provincial capitals and central cities of China. Yangtze River, the world’s third largest river, and its largest tributary, Han River, divide the main city district of Wuhan into three parts, forming the three towns of Wuhan: Wuchang, Hankou, and Hanyang. The geographical location is presented in Figure 1a. The Municipal government planned 17 land-use multifunctional clusters shown in Figure 1b.

Three trajectory data used in this study were GPS data collected from over 10,000 taxis for one ordinary week, including workdays and weekends, from 1 to 7 September in 2009, 19 to 25 September in 2014, and 8 to 14 May in 2015. These data contain the information listed in Table 3: vehicle ID, timestamp, longitude, latitude, and taxi status (0 for vacant, 1 for occupied). Check-in data applied in this study was more than 1 million pieces of Sina Weibo check-in data from 1 May 2013 to 1 May 2015 in Wuhan. This data contains the information listed in Table 4: record ID, check-in time, longitude, latitude, and POI. 

The preprocessing work was to extract PUPs/DOPs. We sorted trajectory data with the same vehicle ID based on timestamps, and extracted PUPs/DOPs according to the change in taxi status. If a taxi with the same vehicle ID changes from vacant status (0) to occupied status (1), then the GPS waypoint recorded at this moment is a PUP; if it changes from occupied status (1) to vacant status (0), then the GPS waypoint recorded at this moment is a DOP. After extraction, we removed ODs where the interval between PUP and DOP was less than one minute. 

### 4.2. Detecting and Evaluating Single-Temporal Urban Clusters

#### 4.2.1. Comparing Detected Results with Planned Clusters

This case study applied the proposed method to detect urban clusters in the year 2014, and finally grouped the divided grid cells into 13 clusters. The transparent grid cells did not group into any cluster since the number of ODs originating from them were less than three (at least three ODs are required to construct a SDE) or removed as outliers during clustering process. In addition, since Dong Lake Scenic District is not a daily activity area, it is not discussed in this article.

**Figure 2:**
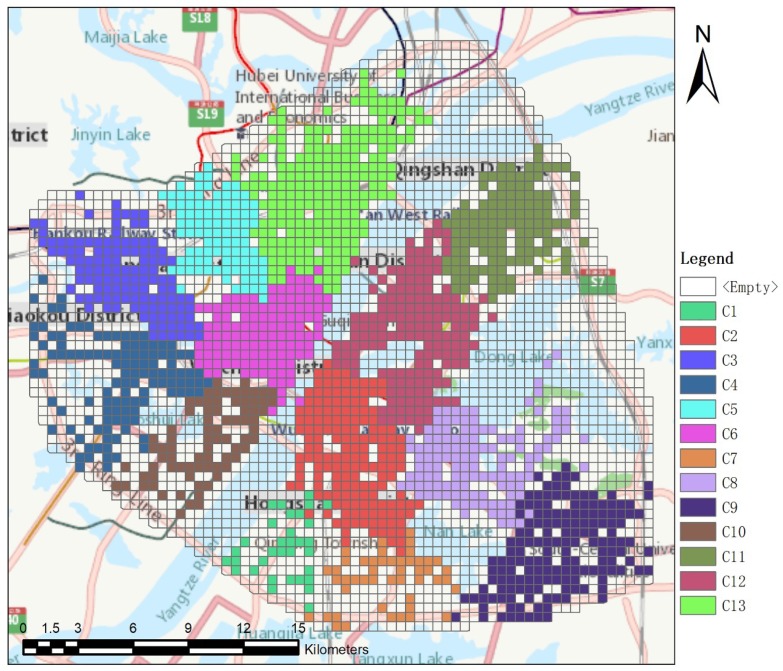
Clustering results in the year 2014.

**Figure 3:**
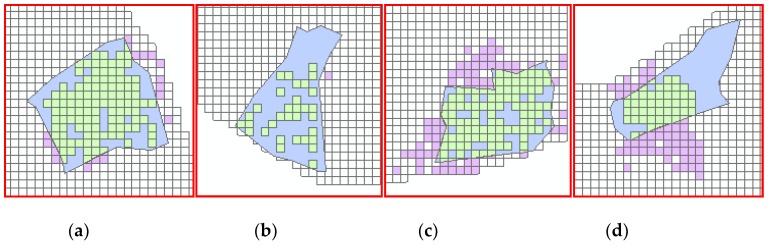
The typical detected clusters for each type in Table 5, in which the purple grid cells represent the detected clusters; the blue polygons represent the corresponding planned clusters; the green grid cells represent the intersections. (**a**) The clustering result of Qingshan (Type 1); (**b**) the clustering result of Baisha (Type 2); (**c**) the clustering result of Guanshan (Type 3); (**d**) the clustering result of Ta Zihu (Type 4).

By comparing Figure 2 with Figure 1b, we find that three clusters, Huangpu, Erqi, and Hanyang Central Activity Zone were not detected. The grid cells where Huangpu is located did not take part in clustering, illustrating that there are few trips starting or ending in those grid cells. A merger between the Erqi or Hanyang Central Activity zone and adjacent clusters reflects that there is a large number of cross-regional travels between them. The 13 detected clusters are classified into four types based on the spatial inclusion relationships between the detected clusters and the corresponding planned clusters shown in Table 5. The typical results from Table 5 are visualized in Figure 3, showing the four types of classification. 

Table 5 shows that there are two detected clusters that agree with the corresponding planned clusters in Type 1. Type 2 suggests that there are four detected clusters smaller than the corresponding planned clusters. Further, the grid cells overlapped by Baisha and Nanhu show sparse distributions like Figure 3b, as there are few trips beginning or ending there, indicating there might be few residents; or these clusters contain lakes, railways, or expressways. This phenomenon reveals that our method can detect urban clusters in more detail. Regarding Type 3, there are four detected clusters, Shisheng, Guanshan, Yangyuan, and Houhu, which are larger than the corresponding planned clusters. In Type 4, there are three detected clusters that deviate from the corresponding planned clusters in some directions, reflecting that some daily travel spaces do not belong to the area of corresponding planned clusters. Through analysis of Table 5 and Figure 3, we qualitatively assessed the progress of urban cluster construction in the year 2014.

#### 4.2.2. Comparing the Differences in Land-Use Functions of the Three Towns of Wuhan

**Figure 4:**
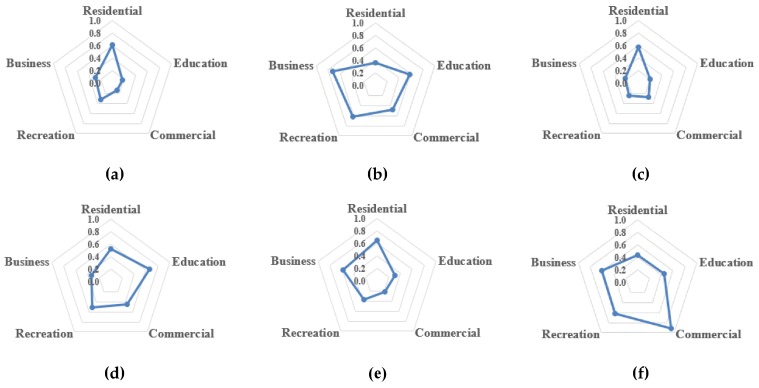

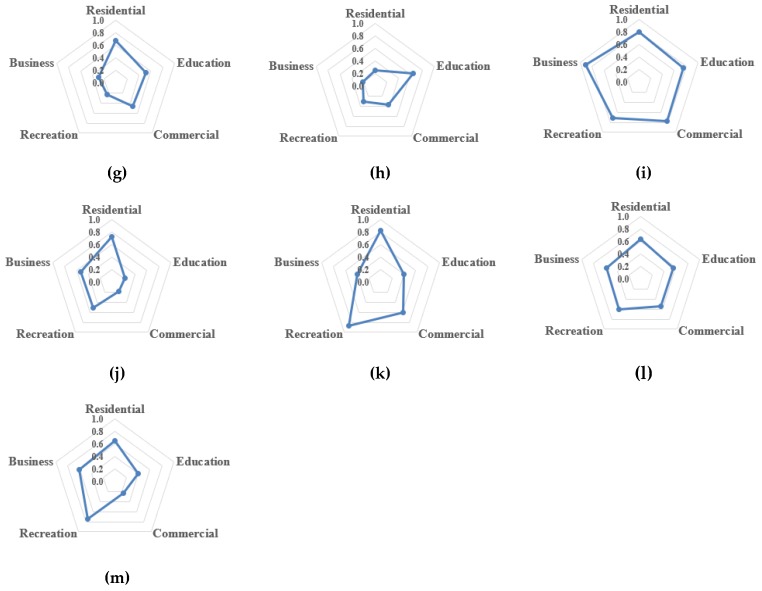
Radar chart of fulfilled demand indexes for cluster (**a**) C1, (**b**) C2, (**c**) C3, (**d**) C4, (**e**) C5, (**f**) C6, (**g**) C7, (**h**) C8, (**i**) C9, (**j**) C10, (**k**) C11, (**l**) C12, (**m**) C13, by five types of land-use functions.

Based on the detected urban clusters, we computed the three indicators shown in Section 3.2.2, to evaluate the rationality of land-use functions in each cluster, in Table 6 and Table 7 and Figure 4. With computed results, we further compared the differences in land-use functions of the three towns of Wuhan from the perspective of a smaller unit, land-use multifunctional cluster, enabling us to investigate land-use patterns of the three regions in greater detail.

The detected clusters in Wuchang relatively agree with planned clusters, but there are still differences in land-use functions among each cluster. Specifically, when comparing Figure 2 with Figure 1b, the number of detected clusters is equal to the planned clusters. But C1 and C8 have an internal travel index below 0.5 in Table 6, suggesting that their internal land-use functions might be poor, leading to a large number of cross-regional travels. Through analysis of Figure 4 and Table 7, we find one interesting phenomenon about C8. The commuting direction index in Table 7 suggests that commuting destinations of residents in Wuchang is mainly toward C8. But Figure 4 shows a low fulfilled demand index for the business function of C8. The analysis seems diametrically opposite, indicating that there might be less residential land but more floating population. In addition, many universities are concentrated there, such as Wuhan University. Hence, although there might be few residents living here, the fulfilled demand index for the education function is still high.

C6 is the activity center of Hankou and Hanyang, with well-developed commercial and business functions. Specifically, Table 6 shows that the main cross-regional travel destinations for C3, C4, C5, C10, and C13 are all toward C6. Figure 4 shows that fulfilled demand indexes for commercial, recreation, and business functions in C6 are more than 0.6, suggesting these functions can fulfill the travel demands, while Table 7 shows that the commuting pattern of C6 is unbalanced—that is, the commuting amount from surrounding clusters to C6 is greater than the amount from C6 to surrounding clusters. These findings are consistent with Hankou Central Activity Zone in the urban plan, which is a site for financial and commercial functions. The plan aims to build up this zone as a modern central business zone with a professional and commercial employment environment [46].

Urban clusters in Hanyang are quite underdeveloped. Only C4 and C10, with a low internal travel index (0.49 and 0.53), are detected in Hanyang with no activity center detected. Furthermore, according to Figure 4, the residential function is the only high fulfilled demand index in C10. The education function in C4 is high but the other indexes are all below 0.6, which indicates that residents need a large number of cross-regional travels to fulfill their demands for other land-use functions. The results suggest that the land-use functions in Hanyang need improvement.

### 4.3. Quantitatively Evaluating Progress of Urban Cluster Construction

**Figure 5:**
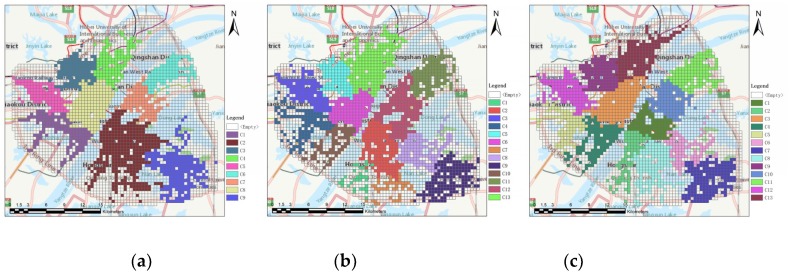
Clustering results in the year 2009 (**a**), 2014 (**b**), and 2015 (**c**) with major highways, including the third ring expressway.

We further applied our method to detect urban clusters in the years 2009 (Figure 5a) and 2015 (Figure 5c). The construction progress of 17 planned clusters can be analyzed based on the three-year clustering results. 

As shown in Figure 5, there are nine detected clusters in Figure 5a, 13 in Figure 5b, and 13 in Figure 5c, indicating that the number of detected clusters is approaching the number of planned clusters. In addition, the number of grid cells participating in the clustering process was 1397, 1563, and 1637 in Figure 5a–c respectively, also showing an increasing trend over time. This phenomenon is particularly evident in the area close to the third ring expressway, as shown in Figure 5. More specifically, several clusters close to the third ring expressway, such as Baisha, Nanhu, Sixin, and Shisheng, gradually formed and approached the shape of the corresponding planned clusters in the period 2009–2015, which suggests that the population of residents living there is increasing. This analysis reflects the spatial expansion of Wuhan.

Based on these three-year clustering results, we further computed *CR* between the detected clusters (Figure 5) and the planned clusters (Figure 1b). In addition, lakes, railways, and expressways are not daily activity spaces, and thus they did not participate in computation. The computed *CR*s were 55.7%, 63.0%, and 69.0% in the years 2009, 2014, and 2015 respectively, suggesting that the detected clusters are gradually approaching the shape of planned clusters on the whole. The calculated *CR*s for the three-year clustering results show the progress of urban cluster construction and indicate the development of land-use functions, which can provide a basis for urban construction.

## 5. Conclusions

In this article, we proposed a DEUC method combining taxi trajectories with Sina Weibo check-in data. In our method, we first constructed weighted SDEs to delineate daily travel spaces, and then applied an AHC method to detect urban clusters based on the similarities in SDEs of each grid cell. We next acquired the demands for land-use functions using a naïve Bayes’ theorem, and adopted three indicators to evaluate the rationality of land-use functions in the detected clusters. Furthermore, we evaluated the conformance between the detected clusters and planned clusters, assessing the progress of urban cluster construction. 

In the case study, urban clusters in Wuhan in the year 2014 were detected and analyzed. The clustering results show that there were 13 detected clusters. By comparing the detected results (Figure 2) with planned clusters (Figure 1b), we found that two of the 13 clusters agreed with the planned clusters, but that the remaining clusters were smaller, larger, or deviated from the planned clusters, qualitatively assessing the progress of urban cluster construction.

By computing the three indicators, we evaluated the land-use functions in each detected cluster, and applied the results in comparing the difference in land-use functions in the three towns of Wuhan from the perspective of a smaller unit. The analysis revealed that detected clusters in Wuchang relatively agreed with planned clusters, but there were still differences in land-use functions among each cluster; Hankou had well-developed commercial and business functions; and only two clusters with low internal travel indexes were detected and no activity center detected in Hanyang, which suggests that land-use functions need improvement.

We further detected urban clusters in the years 2009 and 2015. The three-year clustering results show that the number of detected clusters increased from 9 to 13 in the period 2009–2015, and the computed *CRs* were 55.7%, 63.0%, and 69.0% in the years 2009, 2014, and 2015 respectively, quantitatively assessing the progress of urban cluster construction.

Our results have shown that spatiotemporal big data can provide a new lens for understanding urban clusters in a natural and fine-grained way. Future work will focus on detecting urban clusters using combined trajectory data to assess the progress of urban construction in more detail, which may provide scientific evidence for decision-making in urban development.

## Figures and Tables

**Table 1:** Recent land-use studies with spatiotemporal big data.

Studies	Detection of Functional Zones	Detection of Urban Clusters	Evaluation of the Urban Construction Progress
Soto et al. [12,30]	√		
Pei et al. [28]	√		
Frias-Martinez et al. [31]	√		
Toole et al. [32]	√		
Chen et al. [33]	√		
Caceres et al. [10]	√		
This study		√	√

**Table 2:** Correspondence between POI types in check-in data and resident activity types.

POIs of Check-In Data	Activity Types
Residential locations	In-home
Universities, primary schools, secondary schools...	Schooling
Shopping malls, commercial streets, supermarkets...	Commercial
Cinemas, parks, zoos, museums...	Recreation
Companies, IT companies, financial services companies...	Working

**Table 3:** Examples of taxi GPS data.

Vehicle ID	Timestamp	Longitude	Latitude	Taxi Status
82*****	1411274295	114.*****	30.*****	0
82*****	1411342736	114.*****	30.*****	1
…	…	…	…	…
10*****	1411177122	114.*****	30.*****	1

**Table 4:** Examples of Weibo check-in data.

Record ID	Longitude	Latitude	Check-In Time	POIs
1	114.*****	30.*****	13:42:16	Primary schools
2	114.*****	30.*****	22:03:27	Shopping malls
…	…	…	…	…
10	114.*****	30.*****	20:25:59	Factories

**Table 5:** Classifications of detected clusters.

Type	Category	Corresponding Planned Cluster	Category	Corresponding Planned Cluster
**Type 1** **Detected clusters agree with the corresponding planned clusters.**	C3	Gutian	C11	Qingshan
**Type 2** **Detected clusters are smaller than the corresponding planned clusters.**	C1	Baisha	C7	Nanhu
	C8	Luoyu	C10	Sixin
**Type 3** **Detected clusters are larger than the corresponding planned clusters.**	C4	Shisheng	C9	Guanshan
	C12	Yangyuan	C13	Houhu
**Type 4** **Detected clusters deviate from the corresponding planned clusters.**	C2	Wuchang Central Activity Zone	C5	Ta Zihu
	C6	Hankou Central Activity Zone		

**Table 6:** Cross-regional travel index.

	C1	C2	C3	C4	C5	C6	C7	C8	C9	C10	C11	C12	C13
**C1**	0.42	0.26	0.00	0.01	0.01	0.05	0.14	0.02	0.03	0.01	0.01	0.04	0.01
**C2**	0.04	0.53	0.00	0.01	0.01	0.07	0.08	0.05	0.04	0.01	0.01	0.14	0.01
**C3**	0.00	0.01	0.49	0.16	0.10	0.19	0.00	0.00	0.00	0.01	0.00	0.01	0.02
**C4**	0.00	0.02	0.07	0.49	0.04	0.22	0.00	0.00	0.01	0.11	0.00	0.01	0.02
**C5**	0.00	0.01	0.03	0.02	0.48	0.28	0.00	0.00	0.00	0.01	0.01	0.02	0.12
**C6**	0.00	0.03	0.02	0.03	0.07	0.68	0.01	0.00	0.01	0.04	0.01	0.03	0.08
**C7**	0.05	0.15	0.00	0.00	0.00	0.02	0.54	0.08	0.11	0.00	0.00	0.03	0.01
**C8**	0.01	0.16	0.00	0.00	0.00	0.03	0.11	0.36	0.19	0.01	0.01	0.10	0.01
**C9**	0.00	0.04	0.00	0.00	0.00	0.01	0.05	0.06	0.80	0.00	0.00	0.02	0.01
**C10**	0.00	0.03	0.01	0.09	0.02	0.27	0.01	0.00	0.01	0.53	0.00	0.01	0.02
**C11**	0.00	0.01	0.00	0.00	0.01	0.02	0.00	0.00	0.01	0.00	0.76	0.15	0.03
**C12**	0.01	0.12	0.00	0.00	0.01	0.08	0.02	0.03	0.02	0.01	0.07	0.58	0.06
**C13**	0.00	0.01	0.00	0.01	0.07	0.21	0.00	0.00	0.00	0.01	0.02	0.06	0.59

**Table 7:** Commuting direction index.

	C1	C2	C3	C4	C5	C6	C7	C8	C9	C10	C11	C12	C13
**C1**	--	0.74	0.60	0.54	0.36	0.83	0.59	0.95	0.65	0.45	0.48	0.64	0.56
**C2**	0.26	--	0.22	0.28	0.16	0.51	0.37	0.84	0.48	0.16	0.16	0.26	0.22
**C3**	0.40	0.78	--	0.66	0.40	0.70	0.52	1.00	0.55	0.47	0.50	0.57	0.53
**C4**	0.46	0.72	0.34	--	0.34	0.65	0.51	0.89	0.52	0.44	0.40	0.58	0.42
**C5**	0.64	0.84	0.60	0.66	--	0.78	0.64	0.95	0.73	0.54	0.58	0.63	0.60
**C6**	0.17	0.49	0.30	0.35	0.22	--	0.21	0.74	0.32	0.15	0.23	0.29	0.35
**C7**	0.41	0.63	0.48	0.49	0.36	0.79	--	0.96	0.60	0.46	0.42	0.56	0.52
**C8**	0.05	0.16	0.00	0.11	0.05	0.26	0.04	--	0.07	0.06	0.07	0.22	0.15
**C9**	0.35	0.52	0.45	0.48	0.27	0.68	0.40	0.93	--	0.41	0.35	0.41	0.41
**C10**	0.55	0.84	0.53	0.56	0.46	0.85	0.54	0.94	0.59	--	0.49	0.64	0.57
**C11**	0.52	0.84	0.50	0.60	0.42	0.77	0.58	0.93	0.65	0.51	--	0.54	0.57
**C12**	0.36	0.74	0.43	0.42	0.37	0.71	0.44	0.78	0.59	0.36	0.46	--	0.49
**C13**	0.44	0.78	0.47	0.58	0.40	0.65	0.48	0.85	0.59	0.43	0.43	0.51	--
